# Cultural humility in libraries: a call to action and strategies for success

**DOI:** 10.29173/jchla29863

**Published:** 2025-08-01

**Authors:** Daphne Horn

**Affiliations:** Centre for Addiction and Mental Health Toronto, ON, Canada

Jones SD, Murphy B, editors. **Cultural humility in libraries: a call to action and strategies for success**. Lanham, MD: Rowman & Littlefield; 2024. Hardcover: 179 p. ISBN: 978-1-5381-6214-9. Price: USD$95.00. Available from: https://www.bloomsbury.com/au/cultural-humility-in-libraries-9781538162149/.

*Cultural humility in libraries: a call to action and strategies for success* is a book that is reaching library workers at a critical time. As the editors, Shannon D. Jones and Beverly Murphy, note in their preface, it is “critical to pay attention to social, cultural and political factors that are affecting communities across the United States because these larger movements may be hampering diversity and retention efforts” [1]. There has been no greater political movement to threaten diversity, equity, and inclusion (DEI) work than the Executive Order that President Trump signed in January 2025 that is dismantling DEI policies. The Executive Order targets policies’ removal at federal agencies and federal contractors, while also encouraging the private sector to eliminate their DEI policies [2]. Not just limited to the United States, the ban’s reach extends globally by insisting that others, such as suppliers to U.S. embassies, and countries bidding on U.S. contracts, also comply with the ban [3,4]. With government agencies and companies forced to remove their support for DEI, it is more important than ever for individuals to embark on their own practice of cultural humility.

Cultural humility is an essential framework to DEI policies. While components of it will be challenged in cases where broader DEI policies are dismantled, the framework can still be used to deepen personal practices. There are many different descriptions of cultural humility throughout *Cultural humility in libraries*, however I like the one that Cooke and Hill quote in chapter two and attribute to Foronda et. al from a 2016 article they wrote titled “Cultural Humility: a concept analysis”, published in the *Journal of Transcultural Nursing*. In Cooke and Hill's description of Foronda et al.'s work, there are five attributes that result in persons gaining “mutual empowerment, respect, partnerships and lifelong learning” [5]. The attributes of cultural humility include an openness to new ideas; a self-awareness of one’s own strengths, limitations, and biases; a willingness to put aside one’s ego in order to view everyone respectively; the ability to positively engage with others; and the ability to self-reflect and analyze one’s actions [6]. As Erica Brody and Stacey E. Wahl remind us in chapter five, cultural humility helps achieve the “ultimate goal of librarianship—compassionate, high-quality service for all patrons” [7].

*Cultural humility in libraries* is divided into three parts. Part one consists of four chapters centred around the question of, “what is cultural humility”? This includes a fairly extensive examination of cultural competency, another earlier framework created to address interpersonal and systemic racism. While they are closely linked, one of the differences between the two is that cultural humility has no endpoint. It is a lifelong process of self-awareness, inquiry, and learning [8]. Part two of the book includes six chapters on how to apply cultural humility in the library, and part three has eight chapters where a diverse group of professionals share their personal stories. With over 30 contributors there is a wide range of voices and applications of cultural humility in different settings. While this is important in a book about cultural humility, I felt overwhelmed with the amount of information in the book. In addition, the large number of chapters creates repetition as many of the contributors begin with a definition of cultural humility. A reader could minimize these problems by using the book as a reference text where chapters are read independently, as opposed to reading the book from cover to cover.

In chapter five Brody and Wahl explore ways library staff can expand their knowledge and take action at three levels of cultural humility—personal reflection and learning at an intrapersonal level, working with library patrons at an interpersonal level, and at a collective level tackling systemic problems such as a lack of diversity in the library workforce or hiring practices that do not encourage diversity. At an intrapersonal level Brody and Wahl recommend several actions that readers can take. They encourage us to reflect on explicit and implicit biases, which can be done through tools such as the Implicit Association Test (from Project Implicit) which is available at https://implicit.harvard.edu/implicit/selectatest.html. They also suggest furthering education about cultures different from our own through reading; attending workshops and other programming; watching films and listening to podcasts; and by following diverse groups of people on social media. On an interpersonal level library leadership is encouraged to provide employees with training to learn about and be able to better understand and communicate with the populations that they serve. They also encourage collaborating with different marginalized groups to make decisions about collections and other services. At the collective level Brody and Wahl suggest several initiatives to encourage diversity of the mostly white library and information science profession, including funding to reduce the high cost of tuition, and hiring practices to encourage diversity. Once hired, library workers from marginalized groups need to work in an environment that supports them [7]. The information in this chapter is a microcosm of the book, providing us with ways to apply cultural humility in ourselves, in our work, and in our environment. If you can read only one chapter in the book, I would recommend this one. That does not mean you shouldn’t read the other chapters however, as the book provides resources and inspiration to guide your cultural humility journey.

Chapter seven shares examples of how cultural humility was used to make profound changes at a dental college. In “Advancing cultural humility in dental education,” Lubker and Nelson share how providing professional development through in-person and virtual seminars, creating specialized courses and mentorship for students, and providing library resources for faculty and students, allowed their students, staff, and faculty to learn about cultural humility. The approaches they share can easily be transferred to other health care settings.

Part three of *Cultural humility in libraries* contains personal stories from a diverse group of people. Their stories are often emotional and insightful and allow us to learn from their experiences. As we have discovered, this learning is an important part of cultural humility. This section of the book is exemplified by what Brenda Linares writes as one of eight authors of the chapter, “Beyond the rainbow”. Brenda says, “cultural humility is a critical aspect that we should never forget because we learn from one another. No one can be an expert when it comes to EDI, because each person has his or her own story and experience, and we all need to keep an open mind about who people are” [9].

Cultural humility is a lifelong journey, one where we continue to learn. *Cultural humility in libraries* will provide everyone, from the new library worker to the seasoned professional, the inspiration and knowledge to learn and grow. It is a book that will be read more than once and deserves a spot on your bookshelf.
